# Relationship between upper and lower limb function, cognitive impairment, and depression in patients with chronic obstructive pulmonary disease: A cross-sectional study

**DOI:** 10.1097/MD.0000000000044931

**Published:** 2025-10-17

**Authors:** Jin-Zhuan Zhang, Xiang-Yun Wang, Hong-Jun Ai, Pan-Pan Li, Yuan-Jing Chen

**Affiliations:** a Department of Respiratory Medicine, Kongjiang Hospital, Shanghai, China.

**Keywords:** chronic obstructive pulmonary disease, cognitive decline, depression, Lower Extremity Functional Index, Upper Extremity Functional Index

## Abstract

Chronic obstructive pulmonary disease (COPD) often coexists with limb dysfunction, cognitive decline, and depression. The 20-item Upper Extremity Functional Index (UEFI) and Lower Extremity Functional Index (LEFI) (each 0–80, higher = better) are COPD-specific, yet their links to cognition and mood remain untested. We assessed whether UEFI and LEFI independently predict cognitive impairment and clinically significant depressive symptoms. In this single-center cross-sectional study (January 2021–January 2023), 120 stable out-patients aged ≥40 years with spirometry-confirmed COPD and without confounding neuromuscular disorders were enrolled. Upper and lower limb functions were assessed using the UEFI and LEFI, respectively. Cognitive impairment was defined as a Montreal Cognitive Assessment score < 26; moderate-to-severe depression as a Patient Health Questionnaire-9 score ≥ 10. Both instruments are validated in COPD. Multivariable logistic regression adjusted for age, sex, body mass index, smoking status, forced expiratory volume in 1 second/forced vital capacity ratio, and global initiative for chronic obstructive lung disease stage. Participants (mean age 65.2 ± 7.8 years; 58.3% male) included 35.8% with depression and 29.2% with cognitive impairment. Each 1-standard deviation increase in UEFI reduced the odds of cognitive impairment (adjusted odds ratio: 0.46; 95% confidence interval [CI] 0.31–0.70) and depression (odds ratio: 0.48; 95% CI: 0.30–0.76); corresponding LEFI estimates were 0.65 (95% CI: 0.47–0.90) and 0.51 (95% CI: 0.35–0.74). Associations persisted across sensitivity analyses. Poor self-reported limb function independently predicts cognitive decline and depression in COPD. Incorporating UEFI/LEFI into routine assessment may enable early detection and guide integrated rehabilitation.

## 1. Introduction

Chronic obstructive pulmonary disease (COPD) is a common chronic respiratory disease characterized by progressive airflow limitation, often associated with abnormal airway and alveolar structures.^[1]^ COPD poses a significant global health burden, with high morbidity and mortality rates.^[2]^ As the population ages and risk factors such as smoking persist, the prevalence of COPD is expected to rise.^[3]^

COPD not only affects the lungs but also leads to systemic functional impairments. Patients often experience reduced upper and lower limb function, which impacts their ability to perform daily activities and may further affect cognitive function and mental health.^[4]^ Upper limb function is critical for activities such as dressing and eating, while lower limb function forms the basis for walking and mobility. Studies have shown that muscle strength and endurance in the limbs of COPD patients may be compromised, related to disease severity, physical activity levels, and nutritional status.^[5,6]^ Additionally, COPD patients have higher rates of cognitive impairment and depression compared with the general population, with these symptoms strongly linked to disease prognosis.^[7]^

Previous studies have already demonstrated that COPD-related physical limitations, most commonly assessed by grip strength, gait speed, or 6-minute-walk distance (6MWD), are associated with cognitive decline and depressive symptoms.^[8]^ These studies, however, relied on performance-based or global mobility measures that reflect isolated aspects of limb function. Recent COPD-focused validation studies demonstrate that both the Upper Extremity Functional Index (UEFI)^[9]^ and the Lower Extremity Functional Index (LEFI)^[10]^ were specifically designed to comprehensively quantify the full spectrum of self-reported upper and lower limb activities in daily life. We therefore examined whether UEFI/LEFI scores provide additional insight into cognitive and affective outcomes beyond what has been reported for grip strength, gait speed, or 6MWD. This study aims to evaluate the relationships between upper and lower limb function, cognitive function, and depressive symptoms in COPD patients through a cross-sectional design, providing insights for comprehensive management and rehabilitation.

## 2. Methods

### 2.1. Study design

This cross-sectional study included 120 COPD patients treated at our hospital between January 2021 and January 2023. Inclusion criteria were patients aged ≥40 years with a confirmed COPD diagnosis. Exclusion criteria included active polyneuropathies, inflammatory arthropathies, or primary neuromuscular disorders that could directly impair limb function, ensuring that any observed relationships between UEFI/LEFI scores and cognitive or affective outcomes were not confounded by non-COPD factors.

Sample size was estimated a priori. Assuming a moderate correlation (*R* = 0.30) between limb-function and cognitive scores, 110 participants provided 90 % power (α = 0.05, 2-tailed). Allowing 10 % attrition, we enrolled 120 patients.

This cross-sectional study was approved by the Shanghai Kongjiang Hospital Institutional Review Board (Approval No. 2023021). In accordance with local legislation and institutional requirements, the need for informed consent was waived by the same board because only anonymized, routinely collected data were analyzed.

### 2.2. Data collection

Clinical data were extracted from electronic medical records, including patient demographics (age, sex, body mass index, smoking status), disease characteristics (COPD grading), and pulmonary function test results (forced expiratory volume in 1 second, forced vital capacity, and forced expiratory volume in 1 second/forced vital capacity ratio). Only spirometry performed within 3 months of the functional and cognitive assessments was used; when multiple tests were available, the most recent pre-bronchodilator values were extracted for global initiative for chronic obstructive lung disease (GOLD) staging.

### 2.3. Functional assessments

#### 2.3.1. Upper and lower limb function

The UEFI is a 20-item self-report questionnaire that quantifies difficulty in performing everyday upper limb activities (e.g., reaching, lifting, dressing). Each item is scored 0 to 4 (0 = extreme difficulty/impossible, 4 = no difficulty), yielding a total range of 0–80; higher scores indicate better upper limb function.^[9]^ The LEFI is a parallel 20-item measure of lower limb tasks (e.g., walking, stair-climbing) with identical scoring (0–80); again, higher scores denote better function.^[10]^ UEFI and LEFI were administered as interviewer-guided questionnaires in Mandarin Chinese by trained research nurses. Participants unable to complete an item were instructed to mark “unable to perform” and scored 0; missing items (≤5% of total) were handled with mean imputation at the participant level.

#### 2.3.2. Cognitive and depressive symptoms

Cognitive status was screened with the Montreal Cognitive Assessment (MoCA), a validated 30-point instrument that covers multiple cognitive domains; a score < 26 suggests impairment (one extra point is added for ≤12 years of education).^[11]^ Depressive symptoms were rated with the Patient Health Questionnaire-9 (PHQ-9), a 9-item scale scored 0 to 27; higher scores indicate greater severity (0–4 = minimal, 5–9 = mild, 10–14 = moderate, 15–27 = severe).^[12]^ Both MoCA and PHQ-9 have shown acceptable psychometric properties in COPD populations.

### 2.4. Statistical analysis

Data were analyzed using SPSS 26.0 (IBM Corp., Armonk). Descriptive statistics were calculated for baseline characteristics. Pearson correlation coefficients assessed relationships between UEFI/LEFI and MoCA/PHQ-9 scores. Logistic regression models (univariate and multivariate) evaluated the associations between limb function and cognitive decline/depression, adjusting for potential confounders. *P* < .05 was considered statistically significant.

## 3. Results

### 3.1. Clinical characteristics

A total of 120 COPD patients were included, with a mean age of 65.2 ± 7.8 years and 58.3% male. Most were current or former smokers (92.5%), with a mean body mass index of 26.4 ± 3.2 kg/m^2^. COPD severity distribution was: GOLD I (15.8%), GOLD II (38.3%), GOLD III (30.8%), and GOLD IV (15.0%). Baseline characteristics are detailed in Table 1.

**Table 1:** Clinical characteristics of enrolled patients.

Characteristic	Value
Age (yr)	65.2 ± 7.8
Male	70 (58.3%)
Current/former smokers	111 (92.5%)
BMI (kg/m^2^)	26.4 ± 3.2
GOLD classification	
GOLD I	19 (15.8%)
GOLD II	46 (38.3%)
GOLD III	37 (30.8%)
GOLD IV	18 (15.0%)
FEV_1_ (L)	1.8 ± 0.6
FEV_1_/FVC ratio	0.54 ± 0.1
6MWT distance (m)	375 ± 95
UEFI score	58.4 ± 17.6
LEFI score	49.2 ± 19.1
MoCA score	21.5 ± 4.7
PHQ-9 score	9.3 ± 5.2

Continuous variables compared with independent-samples *t* test or Mann–Whitney *U* test as appropriate; categorical variables with χ^2^ or Fisher exact test.

6MWT = 6-minute walk test, BMI = body mass index, COPD = chronic obstructive pulmonary disease, FEV_1_ = forced expiratory volume in 1 second, FVC = forced vital capacity, GOLD = global initiative for chronic obstructive lung disease, LEFI = Lower Extremity Functional Index, MoCA = Montreal Cognitive Assessment, PHQ-9 = Patient Health Questionnaire-9, UEFI = Upper Extremity Functional Index.

UEFI scores averaged 58.4 ± 17.6, indicating moderate difficulty in upper limb activities. LEFI scores averaged 49.2 ± 19.1, reflecting greater lower limb functional impairment. MoCA scores averaged 21.5 ± 4.7, suggesting mild cognitive impairment. PHQ-9 scores averaged 9.3 ± 5.2, with 35.8% of participants scoring ≥ 10, indicating moderate-to-severe depression.

### 3.2. Limb function in patients with cognitive impairment and depression

**Figure 1.**
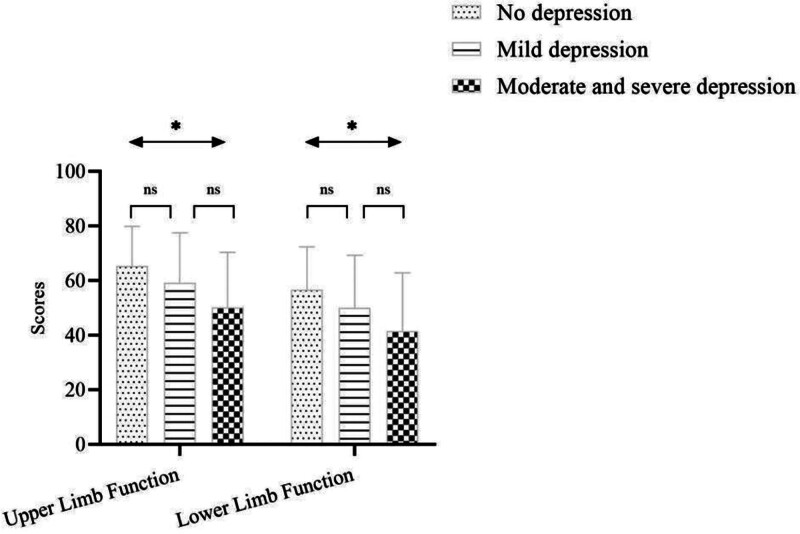
Comparison of upper and lower limb function across depression groups. *: *P* = .01, ns: *P* > .05. Box-and-whisker plots of UEFI (A) and LEFI (B) scores across depression categories (minimal, mild, moderate-to-severe). Horizontal line = median, box = IQR, whiskers = 5 to 95 percentile. Group comparisons: 1-way ANOVA followed by Tukey post hoc test; effect size η^2^ = 0.06 (medium). * indicates *P* < .05 versus minimal depression group. ANOVA = analysis of variance, LEFI = Lower Extremity Functional Index, UEFI = Upper Extremity Functional Index.

Patients with normal cognitive function (MoCA ≥ 26, n = 85) had significantly higher UEFI and LEFI scores than those with cognitive impairment (MoCA < 26, n = 35; *P* = .026 and *P* = .023, respectively) (Table 2). Similarly, UEFI and LEFI scores differed significantly across depression groups (*P* = .01), with the moderate-to-severe depression group showing lower scores than the no-depression group. Figure 1 illustrates UEFI and LEFI scores across 3 PHQ-9-defined depression groups: minimal (0–4), mild (5–9), and moderate-to-severe (≥10). A 1-way analysis of variance showed significant overall differences (UEFI: *F* = 4.92, *P* = .009; LEFI: *F* = 6.31, *P* = .002). Post hoc Tukey tests indicated that participants with moderate-to-severe depression scored markedly lower than those with minimal depression (*P* = .01 for both indices). Thus, poorer limb function is associated with more severe depressive symptoms, highlighting the need to address both physical and mood impairments in COPD management.

**Table 2:** Comparison of upper and lower limb function between patients with normal and impaired cognition.

MoCA score	UEFI score (mean ± SD)	LEFI score (mean ± SD)	Mean difference (95% CI)	Cohen *d* (95% CI)	*P* *
Normal cognition (n = 85)	62.3 ± 15.2	53.7 ± 16.4	UEFI: +7.8 (1.0–14.6)	0.45 (0.08–0.82)	.026
Cognitive impairment (n = 35)	54.5 ± 19.1	44.7 ± 20.8	LEFI: +9.0 (1.4–16.6)	0.47 (0.10–0.84)	.023

Values are mean ± SD. Group comparisons performed with independent-samples *t* test; effect size = Cohen *d* (small ≥ 0.2, medium ≥ 0.5, large ≥ 0.8).

CI = confidence interval, LEFI = Lower Extremity Functional Index, MoCA = Montreal Cognitive Assessment, SD = standard deviation, UEFI = Upper Extremity Functional Index.

* Group comparison: independent-samples *t* test; normality and equal variance assumed (*P* > .05, Shapiro–Wilk and Levene tests). Cohen *d* ≈ 0.5 (medium).

### 3.3. Correlations between limb function, cognitive impairment, and depression

UEFI and LEFI scores showed significant positive correlations with MoCA scores (*R* = 0.62, *P* < .001; *R* = 0.58, *P* < .001) and significant negative correlations with PHQ-9 scores (*r* = −0.72, *P* < .001; *r* = −0.88, *P* < .001). These results indicate that impaired limb function is associated with increased risks of cognitive impairment and depressive symptoms.

### 3.4. Associations between limb function, cognitive impairment, and depression

In fully-adjusted models, each 1-standard deviation increase in UEFI and LEFI was associated with significantly lower odds of cognitive impairment (UEFI: odds ratio [OR] = 0.46, 95% confidence interval [CI] = 0.31–0.70; LEFI: OR = 0.65, 95% CI = 0.47–0.90) and moderate-to-severe depression (UEFI: OR = 0.48, 95% CI = 0.30–0.76; LEFI: OR = 0.51, 95% CI = 0.35–0.74).

These associations persisted across sensitivity analyses using alternative cutoffs for depression and stratification by GOLD stage. When limb function was dichotomized at the cohort mean, higher UEFI (≥58.4) remained significantly associated with lower odds of cognitive impairment (OR = 0.46, 95% CI = 0.25–0.85, *P* = .020) and depression (OR = 0.48, 95% CI = 0.26–0.87, *P* = .017); higher LEFI (≥49.2) was associated with lower odds of depression (OR = 0.51, 95% CI = 0.29–0.94, *P* = .030) but not significantly associated with cognitive impairment (OR = 0.65, 95% CI = 0.35–1.20, *P* = .164).

All sensitivity checks confirmed the direction and approximate magnitude of the associations (Table S1, Supplemental Digital Content, https://links.lww.com/MD/Q282). The fully-adjusted logistic models for cognitive impairment and moderate-to-severe depression are presented in Table 3 and Table 4, respectively.

**Table 3:** Association between limb function and cognitive impairment (MoCA < 26).

Variable	Model 1 OR (95% CI)	*P*	Model 2 OR (95% CI)	*P*	Model 3 OR (95% CI)	*P*
UEFI (per 1 SD ↑)	0.46 (0.31–0.70)	<.001	0.48 (0.32–0.72)	.001	0.46 (0.31–0.70)	.001
UEFI ≥ 58.4 vs <58.4	0.46 (0.25–0.85)	.014	0.48 (0.26–0.89)	.019	0.46 (0.25–0.85)	.020
LEFI (per 1 SD ↑)	0.65 (0.47–0.90)	.009	0.66 (0.48–0.91)	.011	0.65 (0.47–0.90)	.012
LEFI ≥ 49.2 vs <49.2	0.65 (0.35–1.20)	.164	0.67 (0.36–1.24)	.199	0.65 (0.35–1.20)	.164

Model 1: Unadjusted. Model 2: Adjusted for smoking status, FEV_1_/FVC ratio, and GOLD stage. Model 3: Additionally adjusted for age, sex, BMI, smoking status, FEV_1_/FVC ratio, and GOLD stage. The reference category for dichotomized UEFI and LEFI is “lower function” (score < cohort mean).

BMI = body mass index, CI = confidence interval, FEV_1_ = forced expiratory volume in 1 second, FVC = forced vital capacity, GOLD = global initiative for chronic obstructive lung disease, LEFI = Lower Extremity Functional Index, MoCA = Montreal Cognitive Assessment, OR = odds ratio, SD = standard deviation, UEFI = Upper Extremity Functional Index.

**Table 4:** Association between limb function and moderate-to-severe depression (PHQ-9 ≥ 10).

Variable	Model 1 OR (95% CI)	*P*	Model 2 OR (95% CI)	*P*	Model 3 OR (95% CI)	*P*
UEFI (per 1 SD ↑)	0.48 (0.30–0.76)	.002	0.49 (0.31–0.78)	.003	0.48 (0.30–0.76)	.003
UEFI ≥ 58.4 vs <58.4	0.48 (0.26–0.87)	.015	0.50 (0.27–0.92)	.025	0.48 (0.26–0.87)	.017
LEFI (per 1 SD ↑)	0.51 (0.35–0.74)	<.001	0.52 (0.36–0.75)	<.001	0.51 (0.35–0.74)	.001
LEFI ≥ 49.2 vs <49.2	0.51 (0.28–0.93)	.028	0.52 (0.29–0.94)	.030	0.51 (0.28–0.93)	.030

Model 1: Unadjusted. Model 2: Adjusted for smoking status, FEV_1_/FVC ratio, and GOLD stage. Model 3: Additionally adjusted for age, sex, BMI, smoking status, FEV_1_/FVC ratio, and GOLD stage.

BMI = body mass index, CI = confidence interval, FEV_1_ = forced expiratory volume in 1 second, FVC = forced vital capacity, GOLD = global initiative for chronic obstructive lung disease, LEFI = Lower Extremity Functional Index, OR = odds ratio, PHQ-9 = Patient Health Questionnaire-9, SD = standard deviation, UEFI = Upper Extremity Functional Index.

## 4. Discussion

Our cross-sectional study is the first to demonstrate that the UEFI and LEFI, 2 self-report instruments that capture the full spectrum of real-life limb activities, are independently associated with both cognitive impairment (MoCA < 26) and moderate-to-severe depressive symptoms (PHQ-9 ≥ 10) out-patients with COPD.

While correlations do not imply causation, converging evidence suggests that the triad of limb dysfunction, cognitive decline and depression in COPD is mechanistically linked.^[13]^ A growing body of work points to chronic systemic inflammation as a common denominator. Elevated circulating IL-6, TNF-α, and CRP, hallmarks of COPD promote muscle proteolysis via the ubiquitin-proteasome pathway, leading to sarcopenia and reduced UEFI/LEFI scores.^[14]^ Simultaneously, these cytokines cross the blood–brain barrier, impair hippocampal neurogenesis and synaptic plasticity, which manifests as lower MoCA scores.^[15]^ Emerging evidence indicates that contracting skeletal muscle releases myokines such as irisin and cathepsin B, which exert anti-inflammatory and neurotrophic effects on the brain.^[16]^ Poor limb function therefore disrupts this “muscle–brain crosstalk,” depriving the central nervous system of protective signals and accelerating the progression of cognitive and mood disorders.

UEFI and LEFI scores showed significant positive correlations with MoCA scores, suggesting that better limb function is associated with better cognitive function. This aligns with prior research indicating a positive relationship between physical activity and cognitive function.^[17,18]^ Physical activity may enhance cerebral blood flow and oxygen/nutrient supply, benefiting cognitive function.^[19]^ Although the bidirectional link between physical limitation and cognitive decline or depression in COPD is already recognized, our study is the first to demonstrate that the UEFI and LEFI, disease-specific self-report tools, capture this association as strongly as traditional performance measures. Additionally, chronic inflammation and oxidative stress in COPD patients may damage neurons, contributing to both muscle dysfunction and cognitive decline.^[20]^ Physical activity might indirectly improve cognitive function by reducing inflammation and enhancing mood.^[21]^

UEFI and LEFI scores also showed significant negative correlations with PHQ-9 scores, indicating that impaired limb function is linked to more severe depressive symptoms. This is consistent with previous findings that physical functional limitations are associated with depression.^[22,23]^ Reduced physical activity may lead to social isolation and loneliness, increasing depression risk.^[24]^ Multivariate regression analysis further confirmed the independent associations between limb function and cognitive/mental health outcomes, even after adjusting for confounders. Upper-limb limitation compromises basic self-care (e.g., grooming, cooking), while lower limb limitation reduces ambulatory capacity and increases fall risk. These restrictions precipitate activity-related social withdrawal, a key driver of loneliness and depression.^[25]^ Moreover, fear-of-breathlessness during exertion creates a vicious cycle of avoidance behavior, further de-conditioning peripheral muscle and reinforcing negative affect, an observation recently termed the “dyspnea-avoidance hypothesis.”^[26]^

These findings have important implications for COPD clinical management. The present findings argue for routine incorporation of UEFI and LEFI in pulmonary rehabilitation baseline assessments. Because both scales are quick (<5 minutes) and equipment-free, they can be deployed in primary-care or tele-health settings to flag patients at dual risk for cognitive decline and depression.^[27–29]^ Interventions should combine progressive resistance training (targeting lower limb power) and task-specific upper limb training (e.g., unsupported arm exercises) with cognitive-behavioral therapy and goal-oriented coaching to break the dyspnea-avoidance cycle. Longitudinal trials are now warranted to determine whether a 10-point improvement in UEFI or LEFI translates into a clinically meaningful 2- to 3-point increase in MoCA or a 5-point reduction in PHQ-9, as suggested by our logistic models. First, regular assessment of limb function is essential. Second, healthcare providers should consider cognitive and mental health status in COPD treatment and should routinely involve referrals to evidence-based pulmonary rehabilitation and mental-health services. Third, multidisciplinary interventions targeting limb function, cognition, and mental health may improve overall health and quality of life in COPD patients. COPD is inherently syndromic. To isolate COPD-driven limb dysfunction, we excluded active polyneuropathies, inflammatory arthropathies, or primary neuromuscular disorders; this trade-off improves internal validity while limiting external generalizability. Consequently, our findings reinforce, rather than merely suggest, the relevance of evidence-based pulmonary rehabilitation. Incorporating UEFI/LEFI into baseline and serial assessments may help tailor exercise prescription and monitor cognitive-affective response.

Despite these insights, the study has limitations. The cross-sectional design precludes causal inferences, and the single-hospital sample may reflect selection bias. Self-reported UEFI/LEFI without corroborative grip strength, gait speed or 6MWD, and a higher-than-expected prevalence of moderate-to-severe depression. We lacked data on social support, education, and physical-activity volume, and some spirometry, though performed within 3 months, may still have preceded the study visit by several weeks. Future longitudinal studies should validate self-reported UEFI/LEFI against objective performance measures and explore additional contributors such as physical activity, social support, and medication changes.

## 5. Conclusion

In conclusion, this study emphasizes the need for integrated assessment of limb function, cognitive function, and mental health in COPD patients. Early identification and intervention in these domains may enhance quality of life and long-term prognosis. Longitudinal and interventional studies are warranted to determine whether improvements in UEFI/LEFI mediate cognitive and mood gains during pulmonary rehabilitation.

## Author contributions

**Conceptualization:** Jin-Zhuan Zhang, Xiang-Yun Wang, Hong-Jun Ai, Pan-Pan Li, Yuan-Jing Chen.

**Data curation:** Jin-Zhuan Zhang, Xiang-Yun Wang, Hong-Jun Ai, Pan-Pan Li, Yuan-Jing Chen.

**Formal analysis:** Jin-Zhuan Zhang, Xiang-Yun Wang.

**Funding acquisition:** Jin-Zhuan Zhang, Yuan-Jing Chen.

**Investigation:** Hong-Jun Ai, Pan-Pan Li, Yuan-Jing Chen.

**Methodology:** Xiang-Yun Wang, Hong-Jun Ai.

**Supervision:** Hong-Jun Ai.

**Validation:** Yuan-Jing Chen.

**Writing – original draft:** Jin-Zhuan Zhang, Xiang-Yun Wang, Pan-Pan Li, Yuan-Jing Chen.

**Writing – review & editing:** Jin-Zhuan Zhang, Yuan-Jing Chen.

## Supplementary Material


