# Supplementary material for: Relationship between upper and lower limb function, cognitive impairment, and depression in patients with chronic obstructive pulmonary disease: A cross-sectional study
Source: Medicine (Baltimore). 2025 Oct 17;104(42):e44931. doi: 10.1097/MD.0000000000044931 (PMC12537096; doi:10.1097/MD.0000000000044931)
Supplement: Supplementary file 1 [file medi-104-e44931-s001.docx]

**Supplementary Table 1.** **Sensitivity analyses for the association between limb function and depressive symptoms**

| Analysis | Model | UEFI (per 1-SD) β/OR (95 % CI) | P | LEFI (per 1-SD) β/OR (95 % CI) | P |
| --- | --- | --- | --- | --- | --- |
| Continuous PHQ-9 | Linear regression | β = −2.1 (−2.8 to −1.3) | <0.001 | β = −2.4 (−3.2 to −1.6) | <0.001 |
| PHQ-9 ≥ 12 | Logistic regression | OR = 0.43 (0.25–0.73) | 0.002 | OR = 0.49 (0.30–0.78) | 0.003 |
| GOLD II–IV subgroup | Logistic regression | OR = 0.47 (0.28–0.79) | 0.004 | OR = 0.50 (0. |  |

**UEFI: Upper Extremity Functional Index; LEFI: Lower Extremity Functional Index; PHQ-9: Patient Health Questionnaire-9; GOLD: Global Initiative for Chronic Obstructive Lung Disease; SD: standard deviation; OR: odds ratio; CI: confidence interval.**
